# FliC, a Flagellin Protein, Is Essential for the Growth and Virulence of Fish Pathogen *Edwardsiella tarda*

**DOI:** 10.1371/journal.pone.0045070

**Published:** 2012-09-14

**Authors:** Yang He, Tingting Xu, Leif E. Fossheim, Xiao-Hua Zhang

**Affiliations:** College of Marine Life Sciences, Ocean University of China, Qingdao, People’s Republic of China; The University of Plymouth, United Kingdom

## Abstract

*Edwardsiella tarda* is a flagellated Gram-negative bacterium which causes edwardsiellosis in fish. FliC, as a flagellar filament structural protein, is hypothesized to be involved in the pathogenesis of infection. In this study, a *fliC* in-frame deletion mutant of a virulent isolate of *E. tarda* was constructed through double crossover allelic exchange by means of the suicide vector pRE112, and its virulence-associated phenotypes and pathogenicity were tested. It was found that the deletion of *fliC* significantly decreased the diameter of flagella filaments. In addition, the mutant showed reduced pathogenicity to fish by increasing the LD_50_ value for 100-fold compared to the wild-type strain, as well as showed impaired bacterial growth, reduced motility, decreased biofilm formation and reduced levels of virulence-associated protein secretion involved in the type III secretion system (TTSS). The phenotypic characteristics of the *fliC* deletion mutant uncovered in this investigation suggest that *fliC* plays an essential role in normal flagellum function, bacterial growth, protein secretion by TTSS and bacterial virulence.

## Introduction

*Edwardsiella tarda* is an enteric pathogen responsible for significant economic loss in aquaculture with a wide host range including humans [1], and is usually flagellated and motile. The flagellum is an ultrastructure which mediates a number of functions in addition to motility, attachment and chemotaxis. Exhibiting regions of highly homologous amino acid sequences to several proteins of type III secretion system (TTSS), the bacterial flagellum has been considered to serve as a secretory system that might transport various virulence factors [2]. In addition, flagellin was proposed to be a potent activator of innate immune response and thus plays a role either in stimulating host defense or in disease causation [3].

FliC as a flagellar filament structural protein was identified in a variety of organisms. In *E. tarda*, FliC was revealed to be a virulent-strain-specific protein [4], and was further identified as an antigenic protein through the use of rabbit polyclonal antiserum [5]. Purified recombinant FliC showed no apparent immunoprotectivity in a Japanese flounder model when used as a subunit vaccine, yet elicited significantly stronger protective immunity when FliC was fused to other DNA vaccines [6].

Although FliC has been considered associated with virulent strains, no information is available about its exact involvements in the pathogenesis of *E. tarda*. Knock-out of virulence related genes can be used as a strategy to produce attenuated bacterial vaccines [7]. In an attempt to explore the role of FliC as a virulence associated protein, we i) constructed a *fliC* in-frame deletion mutant of *E. tarda* H1 and the corresponding complemented strain; ii) compared the *fliC* in-frame deletion mutant and the wild-type in terms of virulence-associated phenotypes and pathogenicity.

## Results

### Construction of the Δ*fliC* Mutant and the Complementary Strain *fliC* ^+^

Using the double selection strategy of allelic exchange mutagenesis by means of suicide vector pRE112, we deleted 804-bp (residues 166–969) of the *fliC* gene in *E. tarda* H1, thus obtaining the Δ*fliC* mutant with loss of an internal region of the FliC from 56 to 323th amino acid residues. The nonpolar mutant was an in-frame deletion within the open reading frame of the *fliC* gene, and disruption of *fliC* was confirmed by the mutant’s inability to transcript mRNA, which was verified by RT-PCR (data not shown). The Δ*fliC* mutant was confirmed by the ability to grow on TSA supplemented with ColB and inability to grow with Cm. The correct deletion was verified by DNA sequencing of the resulting PCR product.

To confirm that all changes in phenotypes were caused by the deletion of *fliC*, the Δ*fliC* mutant was provided with the intact *fliC* gene in plasmid pACYC184 for complementation analysis. ColB- and Cm-resistant transconjugants were selected, while existence of the plasmid was confirmed by PCR analysis and sequencing.

### FliC is Essential for Flagellum Formation and Motility

**Figure 1:**
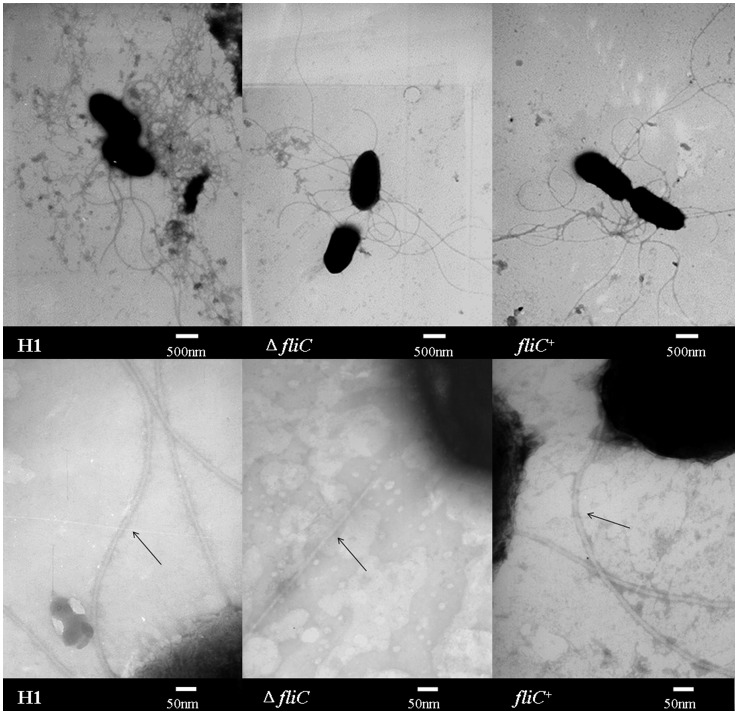
Transmission electron microscopy of *E. tarda* strains. A: Electron micrographs of 10,000× magnification; B: Electron micrographs of 100,000× magnification to show flagella filaments.

Observation with the transmission electron microscope clearly showed that all strains (*E. tarda* H1, Δ*fliC* mutant and *fliC* ^+^) had peritrichous flagella (Fig.1). However, by careful scrutiny of the thickness of 10 flagellar filaments of the strains, we found that the diameters of flagellar filaments produced by the Δ*fliC* mutant were thinner than that of the wild type *E. tarda* and the *fliC* ^+^ (Table1).

**Table 1:** Characterization of *E. tarda* strains.

Parameters	H1	Δ*fliC* mutant	*fliC* ^+^
Diameter of flagella (nm)	13.66±1.09	5.68±0.66**	12.73±0.93
Swarming motility	+	−	+
Swimming motility (swimming plate) (mm)	14.20±2.97	4.10±0.92**	15.70±2.72
Swimming motility (µm/s)	32.28±7.17	14.46±3.27**	36.56±6.10
Biofilm formation (OD_570_)	0.22±0.09	0.16±0.02**	0.20±0.09
LD_50_ (CFU g^−1^)	(1.50±1.70)×10^5^	(1.30±0.90)×10^7^ **	(2.10±1.50)×10^5^

** , P<0.01.

Bacterial motility assays were performed in three different ways. As expected, the Δ*fliC* mutant was drastically impaired in bacterial motility. The swimming motility halos of the wild type H1 and the *fliC* ^+^ tested by swimming plate showed a distinct motile phenotype with larger diffuse spreading diameters than those of the Δ*fliC* mutant (Table 1). The swimming speed directly measured by optical microscopy showed a severe decrease in the Δ*fliC* mutant when compared with the wild type H1 and the *fliC* ^+^ (Table 1), and the ability of direction switching was also hampered in the Δ*fliC* mutant. Furthermore, the Δ*fliC* mutant was almost disabled in the ability to swarm. These results demonstrate that FliC is essential for normal flagellum structure and function to exhibit full motility in *E. tarda*.

### Growth is Dramatically Decreased in the Δ*fliC* Mutant

**Figure 2:**
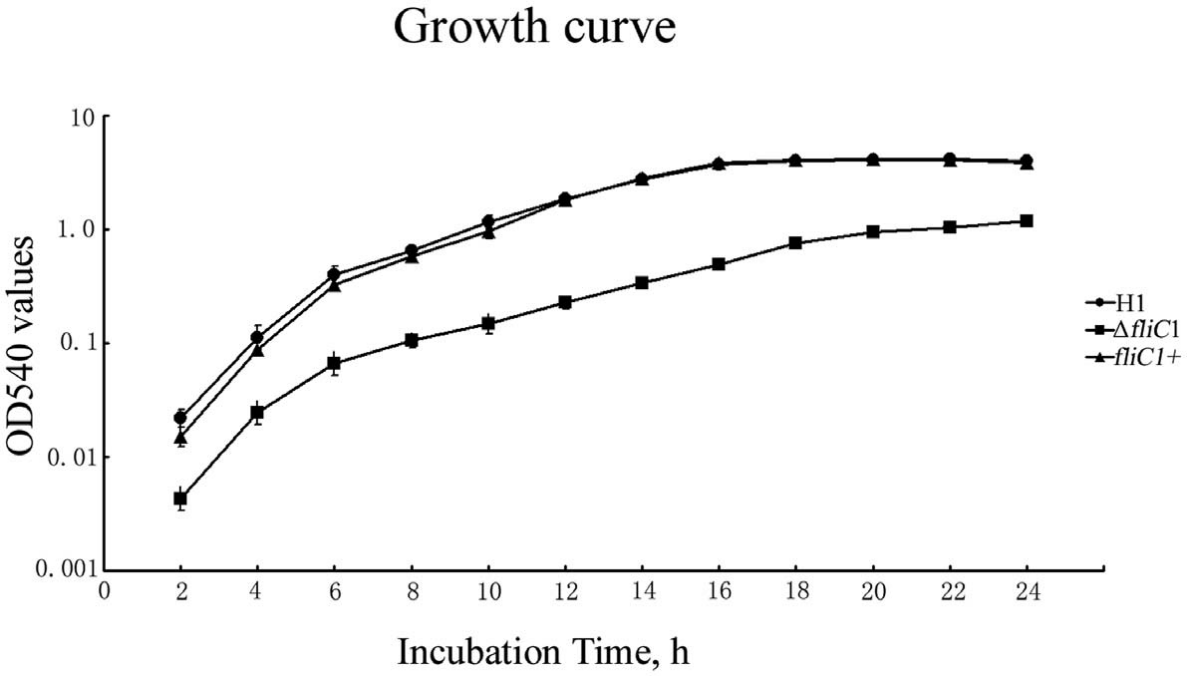
Growth curve of *E. tarda* strains. y axis: OD540 values of *E. tarda* (Log 10); x axis: incubation time.

The Δ*fliC* mutant and *fliC* ^+^ were grown for 30 passages on TSA, and verified by antibiotic selection and PCR, indicating that Δ*fliC* mutant and *fliC* ^+^ were stably maintained. However, in vitro growth kinetics of the Δ*fliC* mutant showed significant growth defect with reduced growth rate and lower maximum cell density when compared with the wild type, whereas the growth of *fliC* ^+^ was almost restored to wild-type levels (Fig. 2). The finding reveals that *fliC* contribute not only to flagellum formation and function, but also to the growth of *E. tarda*.

### *fliC* has Positive Effect on Biofilm Formation

We also examined the role of *fliC* in biofilm formation. The assay was based on the ability of bacteria to initiate biofilm formation on a polystyrene surface. The Δ*fliC* mutant was deficient in biofilm production, producing 27% less than that of the wild type; on the other hand the phenotype was completely restored in the complemented strain, *fliC* ^+^ (Table1), indicating that *fliC* is required for the formation of biofilm in *E. tarda*.

### Protein Secretion of Virulent Secretion Systems Depends on *fliC*

**Figure 3:**
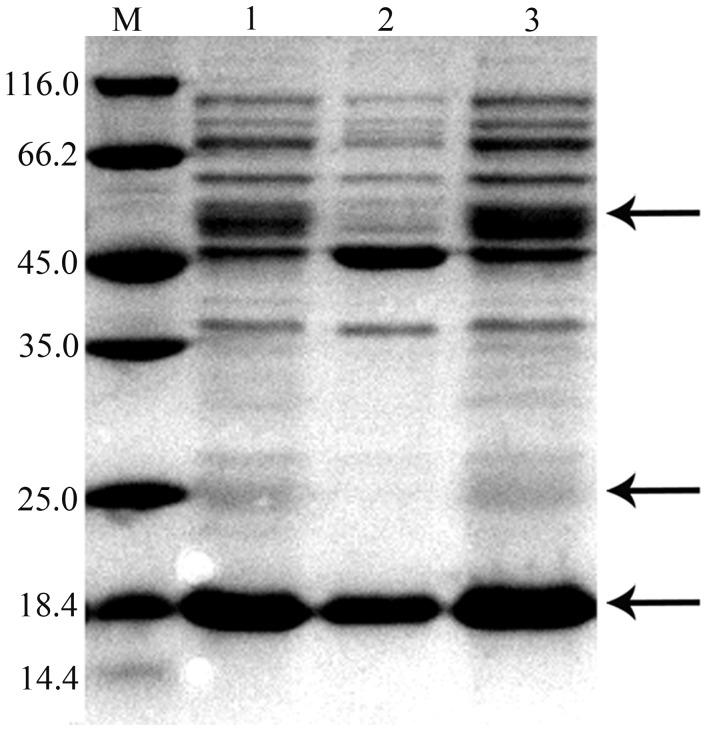
ECP profiles of *E. tarda* strains. M: Protein molecular weight marker, 1: H1, 2: Δ*fliC* mutant, 3: *fliC* ^+^. The major bands are shown by arrows from top: 52 kDa, 22 kDa, 18 kDa.

Flagellum has been reported to mediate the secretion of several extracellular toxins [8]. To determine if the deletion of *fliC* would affect the secretion of ECP in *E. tarda*, extracellular protein profiles of the *E. tarda* strains were surveyed (Fig. 3). ECPs from the culture supernatant of the wild type H1, Δ*fliC* mutant and *fliC* ^+^ were balanced to the same amount, quantifying by an UV spectrophotometer. In general, the Δ*fliC* mutant shared background band profiles similar to those of *E. tarda* H1. However, the Δ*fliC* mutant showed a significant diminishing or complete disappearance of two major bands, approximately 52 and 22 kDa, which were present in *E. tarda* H1. In addition, the 18 kDa band was also attenuated. Comparatively, these protein bands were restored in *fliC* ^+^. Except for the differences in these three protein bands, the samples share similar amount of background bands, indicating the similar extraction efficiency in all samples.

N-terminal sequencing of these proteins was performed and the amino acid sequences obtained were aligned with the deduced protein sequences of *E. tarda* EIB202 on the NCBI website. The N-terminal sequence of the ca. 52 kDa protein was MNNITETRYT, identical to the N-terminal sequence of the TTSS effector protein C, EseC (50.6 kDa). The N-terminal sequence of the ca. 22 kDa protein was TVNTDYHGGG, identical to the N-terminal sequence of the EspA family secreted protein EseB (21.6 kDa), another TTSS effector protein. The N-terminal sequence of the ca. 18 kDa protein was AFDTYIKLDK, identical to the N-terminal sequence of the type VI secretion system (T6SS) protein EvpC (17.8 kDa). Taken together, these results suggest that *fliC* is necessary for the function of secretion systems in *E. tarda*.

### The Δ*fliC* Mutant is Attenuated Virulence for Fish

After verifying the zebra fish (*Danio rerio*) were disease-free [9], it was found that *E. tarda* H1 and the *fliC* ^+^ caused mortalities in zebra fish 1–6 days after being intraperitoneally injected, whereas the death occurred at 3–10 days after infection with the Δ*fliC* mutant. By extending the observing period to 15 days, infection to the slow growing Δ*fliC* mutant was fully established. Both dead and moribund fish exhibited swellings and haemorrhages in the abdomen. Pure cultures of *E. tarda* strains were recovered from the kidney and liver of dead or moribund fish. The Δ*fliC* mutant exhibited a decrease in virulence [LD_50_ value: (1.3±0.9)×10^7^ cfu/g) compared to that of the wild type [LD_50_ value: (1.5±1.7)×10^5^ cfu/g), and the virulence was completely restored in *fliC* ^+^ (Table1). None of the fish in the control group died during the course of the experiment.

## Discussion

FliC has been identified in a variety of organisms as a flagellar filament structural protein, and considered to be functional in the virulence of pathogenic bacteria [10]. However, involvement of *fliC* in bacterial growth or secretion systems has not been reported yet. In this study, *fliC* is found to be required for bacterial growth and the normal function of virulent secretion systems for the first time.

Flagellar filament is helical assembly of repetitive flagellin subunits. Previous work proposed that the C-terminal and N-terminal of the FliC encoding the flagellar filament backbone with a concentric double-tubular structure from ∼1 nm to ∼6 nm in radius, while the middle region form a projection on the surface of the filament, extending out to a radius of ∼12 nm [11], [12]. The Δ*fliC* mutant constructed in this study deleted 804-bp (residues 166–969) in the middle region of the *fliC* gene as a limitation on the length of the known sequence when we started this work. The Δ*fliC* mutant might still be able to assemble into flagella filaments with the remaining C-terminal and N-terminal of the FliC, but becoming thinner for the lack of the projections formed by the middle region of flagellin, which coincide with the previous findings both theoretically and statistically [11], [12]. The remaining FliC has lost its natural conformation, which would affect the normal function of flagellum and thus resulting weakened bacterial motility.

It was interesting to find that there was an essential role of FliC in the growth of *E. tarda*. As flagellum-mediated motility enables the bacteria to migrate toward high-nutrient-concentrated zones and away from toxic substance, flagellum is considered to provide a growth advantage [13], [14]. However, the loss of motility in the Δ*fliC* mutant might not be sufficient to explain the substantial growth defect. The exact role of FliC involved in bacterial growth is currently still unclear and need further investigation.

Biofilm formation is considered to be an important factor in the virulence of pathogenic bacteria [15]. A study in *Burkholderia pseudomallei* has shown that motile flagellum is important for initial attachment and movement required for biofilm formation [16]. Here, the reduced biofilm production of the Δ*fliC* mutant might result from the deficient flagella and less motile ability observed in the Δ*fliC* mutant. In addition, the growth defect of the Δ*fliC* mutant can be another factor for the decrease in biofilm formation.

The Δ*fliC* mutant showed a significant diminishment or complete absence of the three major bands (ca. 18 kDa, 22 kDa and 52 kDa), which were effector proteins in TTSS or T6SS and are generally used by bacterial pathogens to deliver virulence factors into host cells, where they then disrupt a range of cellular functions for subverting normal host cell functions [17]. The secretion of virulence proteins requires a functional flagellar export system and the involvement of flagellar proteins in the secretion of TTSS effector proteins has been proven in the previous studies [8], [18]. Therefore, we deduce that *fliC* is required for the protein secretion of TTSS and T6SS in *E. tarda*.

The TTSS and T6SS, which are conserved among different bacteria, are considered to be key virulence mechanisms of many important gram-negative bacterial pathogens [19]. Mutations and deletions of genes encoding the secretion systems could significantly attenuate the virulence. It was reported that the LD_50_ values of the *eseB* or *eseC* mutants of *E. tarda* were increased by about 10-fold respectively [20], indicating the importance of EseB and EseC in mediating bacterial virulence. Deletion of *evpC* in *E. tarda* led to reduced virulence in blue gourami (*Trichogaster trichopterus*) [21]. As mentioned above, the Δ*fliC* mutant showed significantly attenuated virulence, which might be partially attributed to the loss of TTSS effector proteins (EseB and EseC) and the T6SS effector protein EvpC, which could be involved in pathogenesis of *E. tarda*. In addition, the observed defects in bacterial growth, motility and biofilm formation might correlate with one another and collaborate in attenuating the virulence of the Δ*fliC* mutant.

In conclusion, the phenotypic characteristics of the Δ*fliC* mutant uncovered in this investigation demonstrate that *fliC* is not only critical for flagellum formation, motility, bacterial growth and biofilm formation, but also important to the normal function of secretion systems for virulence proteins and bacterial virulence. The restoration of these phenotypes in the complementary strain confirms the involvement of *fliC* in these functions. Taken together, the diverse roles of *fliC* in *E. tarda* during infection indicate its significance in promoting virulence.

## Materials and Methods

### Bacterial Strains, Plasmids and Growth Conditions

Bacterial strains and plasmids used in this study are described in Table 2. *E. tarda* was grown in Tryptic-Soy broth (TSB) or on Tryptic-Soy agar (TSA) at 28°C, while *Escherichia coli* was cultured in Luria broth (LB) at 37°C. When required, appropriate antibiotics were added at final concentration of: colistin B (ColB, 125 µg ml^−1^); and chloramphenicol (Cm, 50 µg ml^−1^) (Solarbio). The authenticity of the bacterial culture was verified by 16S rRNA gene sequencing.

**Table 2:** Bacterial strains and plasmids used in this study.

Strains or plasmids	Characteristics	References or sources
*Edwardsiella tarda*		
H1	Pathogen isolated from a mariculture farm in Wenden, China. Col^r^, Tc^r^	[22]
Δ*fliC*	H1, in-frame deletion of *fliC*	This study
*fliC* ^+^	H1, Δ*fliC* complementation with intact *fliC* gene	This study
*Escherichia coli*		
SY327 (λpir)	Δ (*lac pro*) *argE*(*Am*) *rif malA recA*56 *rpoB* λ *pir*, host for π-requiring plasmids	Umeá University
S17-1 (λpir)	*Tpr Smr recA thi pro rK- mK- RP4∶2-Tc:MuKm Tn7 λ pir (thi pro hsdR hsdM+ recA RP4-2-Tc : Mu-Km-Tn7*)	Umeá University
Plasmids		
pUCm-T	Cloning vector, Amp^r^	Sangon, Shanghai
pUCmD*fliC*	pUCm-T derivative containing *fliC* bp1–165 fused in-frame to bp970–1251, Amp^r^	This study
pRE112	pGP704 suicide plasmid, pir dependent, oriT, oriV, sacB, Cm^r^	[23]
pRE112D*fliC*	pRE112 derivative containing *fliC* bp1–165 fused in-frame to bp970–1251, Cm^r^	This study
pACYC184	Cm^r^, Tc^r^	Fermentas Life Sciences
pACYC184+*fliC*	pACYC184 derivative containing 1.4 kb fragment of *fliC* putative promoter and ORF, Cm^r^	This study

### Construction of *fliC* in–frame Deletion Mutant (Δ*fliC* mutant)

The nucleotide sequence of *fliC* (ETAE_2130) was provided by professor Yuan-Xing Zhang, East China University of Science and Technology. A *fliC* in-frame deletion (Δ*fliC*) mutant of *E. tarda* H1 was constructed by double crossover allelic exchange using suicide vector pRE112 [23]. In brief, the DNA fragment containing the Δ*fliC* was amplified using primers (*fliC*-UO, *fliC*-UI & *fliC*-DI, *fliC*-DO) shown in Table 3. A DNA fragment containing 165 bp of the 5′ end of *fliC* and 199 bp upstream of the ATG initiation codon was amplified from chromosomal DNA by PCR using primers *fliC*-UO and *fliC*-UI. A DNA fragment containing 282 bp of the 3′ end of *fliC* and 2 bp downstream of the TAA stop codon was amplified using primers *fliC*-DI and *fliC*-DO. Both fragments were purified and fused in a subsequent PCR reaction using primers *fliC*-UO and *fliC*-DO. The fused segment (Δ*fliC*) was sequenced and then ligated into pRE112, and the resulting plasmid pRE112Δ*fliC* was transformed into *Esc. coli* SY327 (λpir), which is readily transformable but lacks the *tra* genes of RP4 [24]. The plasmid was then introduced into *Esc. coli* SY17-1 (λpir) for mobilization into *E. tarda* H1 by conjugation. The transconjugants containing plasmid pRE112Δ*fliC* integrated into *E. tarda* H1 chromosome by a single crossover event were selected on TSA containing Cm and ColB. Allelic exchange between the chromosomal gene and the mutagenized plasmidic copy was achieved by the second crossover event and was counter-selected on TSA containing 10% sucrose to determine the excision of pRE112 from the chromosome. The resultant strain, Δ*fliC* mutant, was selected by antibiotic (Cm) sensitivity, and was confirmed by PCR with *fliC*-U & *fliC*-D and sequencing.

**Table 3:** Primers used for *fliC* deletion and complementation.

Primers	Sequences (5′–3′)
*fliC*-UO	AA**TCTAGA**CGATGGGTCAATAGAAGCA^a^
*fliC*-UI	CGGCGGTCTTAACGGTGAAGCGGTTGGAGA^b^
*fliC*-DI	ACCGTTAAGACCGCCG ATC
*fliC*-DO	AA**TCTAGA**GATTAACGCAGCAGAGAC
*fliC*-U	CCC**AAGCTT**CGATGGGTCAATAGAAGCA
*fliC*-D	CG**GGATCC**GATTAACGCAGCAGAGAC

a Nucleotides in bold represent restriction enzyme sites added to the 5′ region of the primer.

b Nucleotides underlined represent 15-bp overlap sequences.

### Construction of Complementary Strain (*fliC* ^+^)

To construct a complementary strain of the Δ*fliC* mutant, an intact *fliC* gene containing the putative promoter region was amplified with primers *fliC*-U and *fliC*-D. The PCR fragment obtained was cloned into *HindIII* and *BamHI*-digested pACYC184 to construct pACYC184+*fliC*. The constructed plasmid was electroporated into the Δ*fliC* mutant strain to produce *fliC* ^+^. Col and Cm resistant transconjugants were selected, and presence of the plasmid was confirmed by PCR analysis and sequencing.

### Electron Microscopy and Motility Assay

*E. tarda* strains of overnight cultures were negatively stained with 1% phosphotungstic acid (pH 7.4) on a Formvar carbon- coated grid [25] and observed with a transmission electron microscope (TEM-1200EX, Japan). Swimming motility was assessed with TSB plates containing 0.3% (w/v) agar inoculated with a sterile toothpick, and swimming distance was measured as the distance from the point of inoculation to the edge of bacteria in the plate. Swarming motility was assessed with TSB plates consisted of 0.5% (w/v) agar and 5% (w/v) glucose as describe by Rashid and Kornberg [26]. Bacterial motility was also observed directly by microscopy and swimming speeds were measured on a screen from frames at 200 ms interval [27]. Each experiment was carried out in triplicate.

### Growth Determination

Overnight cultures were prepared for all strains (*E. tarda* H1, Δ*fliC* mutant and *fliC* ^+^). The growth densities of these cultures (based on OD540 readings) were then equalized by dilution adjustments. Following this, the equalized cultures were further diluted 100-fold into TSB, where upon growth with shaking began. The OD540 was measured every 2 hours until the bacterial growth began to decrease.

### Quantitative Biofilm Formation Assay

The biofilm assay was executed according to a previously described procedure [28] for three times. Briefly, overnight cultures of *E. tarda* grown in TSB medium were adjusted to 0.5 of OD_540_ and were diluted 1∶20 in fresh TSB. The cultures (200 µl) were transferred into wells of a 96-well microtiter plate and were incubated for 2 days. The samples were stained with 2% crystal violet for 15 min after washing with water and methanol fixation. The biofilm was quantified by an ELISA reader (SUNRISE™, Tecan Group Ltd.) at 570 nm after the samples were resolubilized in 150 µl of 95% ethanol.

### Extracellular Product (ECP) Preparation and Sequencing Analysis

The ECP of pathogenic bacteria contains various bio-molecules, some of which are responsible for bacterial virulence [29]. The ECPs of the *E. tarda* strains were prepared as described by Abbass *et al*. [30]. One-dimensional (1D) sodium dodecyl sulfate–polyacrylamide gel electrophoresis (SDS-PAGE) of the extracellular protein contents were then performed according to standard procedures. For sequencing analysis, proteins were transferred to PVDF membrane with a semi-dry system (EPS601), and Edman N-terminal sequencing of the proteins was performed with a Procise model 491 protein sequencer (Applied Biosystems).

### Test of Bacterial Virulence in Fish Model

The zebra fish (*Dario rerio*) used for virulence tests in this study are cultured animals, and all the experiments are programmed in strict accordance with the regulation of local government. The Animal Ethics Committee of Shandong Province, China has approved this study.

Zebra fish (average weight of 0.3 g) from quarantined stocks were acclimatized for more than one week in the laboratory. These animals were recognized as disease-free and used as models to assess the virulence of *E. tarda* strains (H1, Δ*fliC* and *fliC* ^+^). Each group of 10 fish were infected intraperitoneally with 20 µl PBS-washed bacterial cells which were adjusted to the required concentrations of 10^3^–10^7^ CFU ml^−1^ and the bacterial numbers were determined by direct counting using hemocytometer [31]. The control groups were injected with 20 µl PBS. The fish were maintained in static fresh water (50% of the volume was changed daily) at 20°C over a period of 15 days. The LD_50_ values were calculated as described by Wardlaw [32].

### Statistical Analysis

Statistical analysis was performed using SPSS. Paired t-test was conducted for comparison between the wild type, the Δ*fliC* mutant and the complementary strain *fliC* ^+^. Values were expressed as the mean±SD from three independent experiments, and P-value<0.01 was considered statistically significant.
